# Defective phosphatidylethanolamine biosynthesis leads to a broad ataxia-spasticity spectrum

**DOI:** 10.1093/brain/awaa442

**Published:** 2021-01-17

**Authors:** Rauan Kaiyrzhanov, Saskia Wortmann, Taryn Reid, Mohammadreza Dehghani, Mohammad Yahya Vahidi Mehrjardi, Bader Alhaddad, Matias Wagner, Marcus Deschauer, Isabell Cordts, J Pedro Fernandez-Murray, Veronika Treffer, Zahra Metanat, Alan Pittman, Henry Houlden, Thomas Meitinger, Christopher Carroll, Christopher R McMaster, Reza Maroofian

**Affiliations:** 1 Department of Neuromuscular Diseases, University College London, Queen Square, Institute of Neurology, London, UK; 2 Institute of Human Genetics, Technical University Munich, Munich, Germany; 3 Institute of Human Genetics, Helmholtz Zentrum Munich, Neuherberg, Germany; 4 Radboud Center for Mitochondrial Medicine, Department of Pediatrics, Amalia Children’s Hospital, Radboudumc, Nijmegen, The Netherlands; 5 University Childrens Hospital, Paracelsus Medical University (PMU), Salzburg, Austria; 6 Department of Pharmacology, Dalhousie University, Halifax, Canada; 7 Medical Genetics Research Center, Shahid Sadoughi University of Medical Sciences, Yazd, Iran; 8 Abortion Research Centre, Yazd Reproductive Sciences Institute, Shahid Sadoughi University of Medical Sciences, Yazd, Iran; 9 Department of Neurology, Technical University of Munich, School of Medicine, Munich, Germany; 10 Provincial Clinical Genetic Counselling Center, Zahedan University of Medical Sciences Zahedan, Iran; 11 Genetics Centre, Molecular and Clinical Sciences Institute, St George's University of London, London, UK

With great interest, we read the article by Rickman *et al.* (2020), highlighting the link between defective phosphatidylethanolamine (PE) biosynthesis and hereditary spastic paraplegia (HSP). The cytidine diphosphate (CDP)-ethanolamine pathway is a three-step enzymatic cascade involved in PE biosynthesis. The rate-limiting enzyme in this pathway, ethanolamine-phosphate cytidylyltransferase (ET), is encoded by *PCYT2* (OMIM 618770), and biallelic variants in this gene have been associated with a clinical spectrum of HSP (Vaz *et al.*, 2019; Vélez-Santamaría *et al.*, 2020). Selenoprotein 1 (*SELENOI*) (OMIM 607915, also known as EPT1), catalyses the final step in the biosynthesis of PE via the CDP-ethanolamine pathway (Horibata and Hirabayashi, 2007). Biallelic variants in *SELENOI*/EPT1 have also been linked to complex HSP in two families (Ahmed *et al.*, 2017; Horibata *et al.*, 2018). Here, we report two additional individuals harbouring biallelic variants of *PCYT2* and *SELENOI*/EPT1.

Case 1 was a female, who was born as a second child to healthy, non-consanguineous German parents after an unremarkable pregnancy and birth. Her older sister died in adulthood of probable myocarditis. The affected individual failed to thrive, and at the age of 3 years she was noted to have severely reduced vision. At 5 years of age, early-onset severe retinal dystrophy (EOSRD) was diagnosed. In addition, bilateral cataracts were observed, and these were treated surgically at the age of 6 years. Epilepsy with mostly generalized tonic-clonic seizures was diagnosed at 13 years of age, and EEG showed occipital lobe epileptiform discharges. Progressive bilateral hearing loss was reported beginning at age 19 years and attributed to sensorineural damage. A muscle biopsy at age 14 years was normal (no signs of mitochondrial disease on immunohistochemistry). An intellectual impairment was not observed (total IQ at low normal range, with a selective lower value for ‘word fluid’) and the patient embarked on a university degree. At the age of 16 years, the patient complained of gait abnormalities and a physical examination showed signs of cerebellar ataxia with spontaneous nystagmus, ataxic gait and limb ataxia. Although hyperreflexia was noticeable, no spasticity was observed, and the Babinski sign was negative. No muscle weakness or dysmorphic features were detected. MRI of the brain demonstrated cerebellar atrophy (Fig. 1G) and that of the spine showed atrophy of the spinal cord predominantly at the thoracic level (Fig. 1G and Supplementary Fig. 2). The latency of the sensory evoked potential (P40) of the tibial nerve was delayed to 53 ms on the right side; no potential was present on the left side. At the age of 28 years, she was found dead in the bathtub, presumably after a seizure.

**Figure 1:**
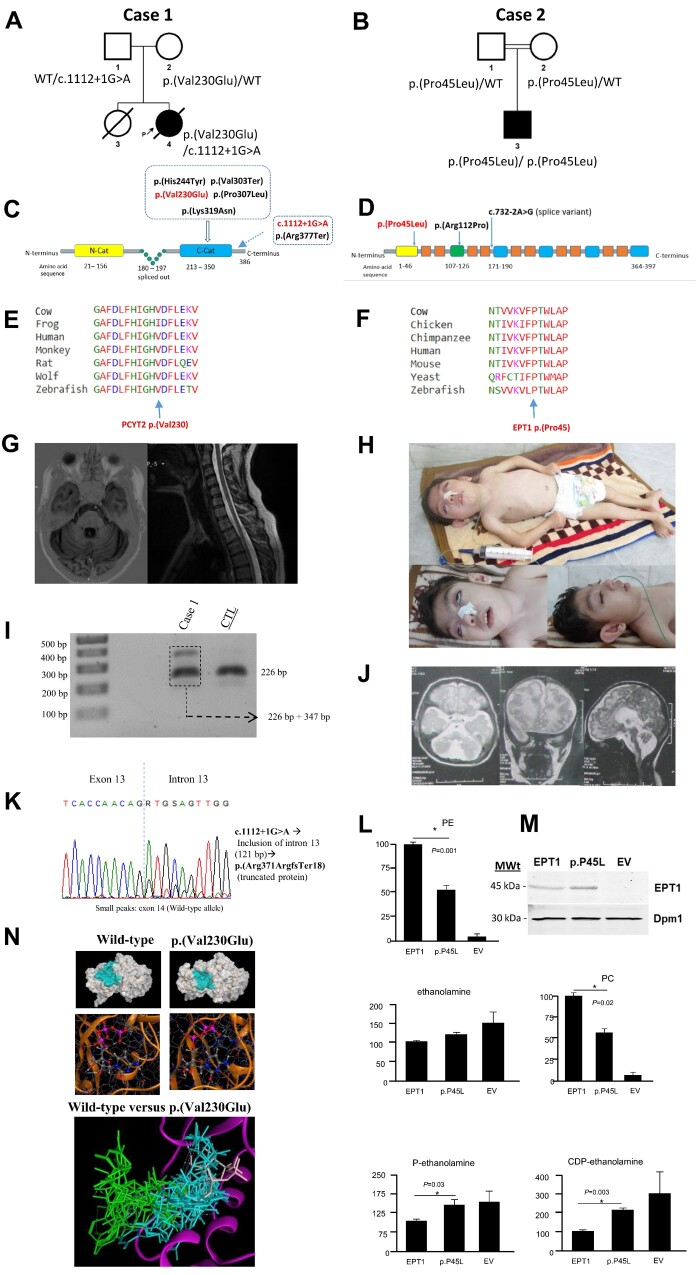
**Genetic and clinical summary of the investigated cases with functional characterization of *SELENOI*/EPT1 p.(Pro45Leu) deﬁciency and molecular modelling of the *PCYT2* p.(Val230Glu) variant.** (**A**) Family tree for Case 1. Square = male; circle = female; filled symbol = affected individual; open symbols = unaffected carriers; WT = wild-type allele. (**B**) Family tree for Case 2. Square = male; circle = female; filled symbol = affected individual. The double line indicates consanguinity. (**C**) Domain structure of PCYT2β with localization of the disease-associated variants. PCYT2β splicing results in the deletion of residues 180–197 from the central linked segment (Pavlovic *et al.*, 2014). PCYT2 protein is predicted to have two catalytic domains. The yellow and blue box indicate the N-catalytic domain and C-catalytic domain, respectively. Pathogenic variants reported in previous papers and identiﬁed in this study are shown in black and red, respectively. (**D**) The predicted transmembrane and cytoplasmic domains of the SELENOI/EPT1 protein (Q9C0D9). The SELENOI/EPT1 protein (NM_033505.3) contains 397 amino acid residues spanning the predicted six cytoplasmic and 10 transmembrane domains. The yellow box indicates the endoplasmic reticulum luminal facing domain. The green box shows the CDP-alcohol phosphotransferase domain. Blue boxes illustrate other cytoplasmic domains and orange boxes show transmembrane domains. Pathogenic amino acid changes reported in previous papers and identiﬁed in this study are shown in black and red, respectively. (**E**) Protein multiple sequence alignment showing that p.Val230 is located in the highly-conserved PCYT2 protein region. (**F**) Protein multiple sequence alignment showing that p.(Pro45) is located in the highly-conserved SELENOI/EPT1protein region. (**G**) Brain and cervical/thoracic spinal cord MRI images from Case 1. The T_1_-weighted axial scan of the brain on the *left* shows cerebellar atrophy. The T_2_-weighted sagittal scan of the spinal cord on the *right* shows spinal cord atrophy at the level of the thoracic segment. (**H**) Clinical photos from Case 2. The *top* image demonstrates a profoundly disabled patient with short and malnourished stature, distal and proximal flexion contractures in upper limbs, hyperextension of the knees and external deviation of the feet. The *bottom* images show tonic upgaze, dysmorphic facial features such as large protruding ears, hypertelorism and a wide nasal bridge. Feeding occurs through the nasogastric tube. (**I**) Gel electrophoresis from cDNA analysis of the *PCYT2* c.1112+1G>A variant showing two forms of *PCYT2* splice products of 226 and 347 bp, respectively in Case 1. (**J**) Brain MRIs from Case 2 showing widespread T_2_-weighted white matter hyperintensities with cerebral and cerebellar atrophy on the left and middle images. The image on the right depicts brainstem and cerebellar atrophy. (**K**) Electropherogram showing that *PCYT2* c.1112+1G>A splice variant results in the retention of intron 12 and the introduction of a premature stop codon after 17 amino acids. (**L**) Incorporation of radiolabelled ethanolamine into the metabolites of the CDP-ethanolamine pathway. Yeast cells that had their endogenous *EPT1* gene inactivated were transformed with a plasmid expressing human *SELENOI*/EPT1, and *SELENOI*/EPT1 p.(Pro45Leu) and an empty vector (EV). Cells were incubated with radiolabelled ethanolamine for 1 h, the metabolites of the CDP-ethanolamine pathway were separated by thin-layer chromatography and the incorporation of radiolabel into each CDP-ethanolamine pathway metabolite was determined by scintillation counting. (**M**) Western blot showing the expression of human SELENOI/EPT1 and SELENOI/EPT1 p.(Pro45Leu) in yeast cells. (**N**) Protein modelling predicts that the PCYT2 p.(Val230Glu) variant affects the overall structure of the substrate binding pocket and has a strong negative effect on binding of CTP (shown) and phosphoethanolamine (not shown) to the active site of PCYT2. Blue indicates CTP binding to wild-type; green indicates CTP binding to p.(Val230Glu).

Exome sequencing on the proband’s DNA was performed as previously described (Wagner *et al.*, 2019). Two heterozygous variants in *PCYT2* (NM_001184917.2: c.1112+1G>A; and c.743T>A, p.(Val230Glu) were identified. Sanger sequencing confirmed both variants in the index and carriership of one variant each in both parents (Fig. 1A and Supplementary Fig. 1). The canonical splice variant was absent from the Genome Aggregation Database (gnomAD), Queen Square Genome database of 16 000 exomes and the Munich in-house database of 21 000 exomes (Supplementary Table 1). *In silico* tools strongly predicted an alteration of splicing (Human Splicing Finder: −165%). Subsequent cDNA analysis conducted as previously described (Krenn *et al.*, 2019) from fibroblast RNA revealed the presence of an additional longer splice product in comparison with a control sample on gel electrophoresis (Fig. 1I). It should be noted, however, that this experiment did not exclude the expression of correctly spliced RNA from the respective allele. Subsequent Sanger sequencing of the two bands revealed that the splice variant resulted in the retention of intron 12 and introduction of a premature stop codon after 17 amino acids (Fig. 1K).

The missense variant was absent from our in-house databases and seen only once in a heterozygous state in 251 240 gnomAD alleles. *In silico* tools consistently predicted the missense variant to be deleterious (PolyPhen-2: 0.987, SIFT: 0, CADD: 32, M-CAP: 0.644) (Supplementary Table 1). This altered amino acid was highly conserved (Fig. 1E) and located within the second cytidylyltransferase (CTP) catalytic domain of PCYT2 (Fig. 1C) (Pavlovic *et al.*, 2014) immediately adjacent to the p.His226X227Gly228His229 catalytic motif. Molecular Operating Environment version 2019.0102 using the Amber10: EHT force field was used for molecular dynamic simulation to predict the effect of p.(Val230Glu) on PCYT2 structure and function. The ligand binding pocket of p.(Val230Glu) was less open than the wild-type and resulted in CTP being far less likely to dock in its normal binding site in the p.(Val230Glu) variant. In addition, there were alterations between preferred amino acid residue binding within the catalytic site for both CTP and phosphoethanolamine for the p.(Val230Glu) variant compared with the wild-type enzyme (Fig. 1N). The p.(Val230Glu) change was immediately adjacent to the PCYT2 catalytic motif and molecular dynamics modelling predicted deficient substrate binding for the p.(Val230Glu) variant.

Case 2 was a 5-year-old male, who was born full-term to consanguineous parents of Balochi ethnicity from Iran. He was born after an unremarkable pregnancy with a below-average birth weight and occipitofrontal circumference (15th percentile). The disease manifested though remarkable growth failure and profound psychomotor retardation. He failed to sit or ambulate independently or develop speech and non-verbal communication skills. From the early postnatal period, he developed daily recurring generalized tonic-clonic seizures and paroxysmal tonic upgaze. Phenobarbital significantly reduced the frequency of the seizures to one per week, and he has been seizure-free for the last 4 months. Because of the lack of growth and prominent feeding difficulties, his current body mass index is 11.2 (the 1st percentile). Additionally, he had severe microcephaly, scoliosis and congenital cataract. There are signs of facial dysmorphism including large, low-set protruding ears, hypertelorism and a wide nasal bridge (Fig. 1H).

His neurological examination revealed bilateral visual impairment, paroxysmal tonic upgaze and dysphagia together with central hypotonia, severe appendicular spasticity and distal and proximal flexion contractures in the upper limbs (Supplementary Video 1). In addition, there was hyperextension of the knees, external deviation of the feet, brisk tendon reflexes, reduced power, and wasting of muscles (Fig. 1H). While his metabolic screening was unrevealing, brain MRI showed cerebral hemispheric, pontine, medullary and cerebellar atrophy with extensive hypomyelination and ventriculomegaly (Fig. 1J). EEG showed bilateral posterior abnormalities in the context of artefactual activity. Electrophysiology studies and ophthalmological examination were not available.

To identify the genetic cause of the disease in the affected individual, exome sequencing on DNA extracted from the proband’s leucocytes and variant filtering were performed as previously described (Makrythanasis *et al.*, 2018). A novel homozygous missense variant in exon 3 of *SELENOI*/EPT1 c.134C>T, p.(Pro45Leu) (NM_033505.3) residing within a 23.7 Mb region of homozygosity was identified. The p.(Pro45Leu) variant was located in a highly conserved SELENOI/EPT1 protein in the N-terminal region of an endoplasmic reticulum luminal-facing domain (CADD score of 26.9 and high conservation GERP score of 5.96) (Fig. 1D and F). The variant was absent from gnomAD and predicted to be probably damaging (PolyPhen-2) or deleterious/damaging (PROVEAN) (Supplementary Table 1).

To determine the functional significance of the variant, we assessed the effect of p.(Pro45Leu) on SELENOI/EPT1 activity in a yeast strain (Supplementary material). Cells expressing SELENOI/EPT1 p.(Pro45Leu) displayed a significant increase in radiolabel associated with CDP-ethanolamine compared with wild-type *SELENOI*/EPT1and a decrease in radiolabel associated with the downstream lipids PE and phosphatidylcholine (Fig. 1L). This was consistent with an impairment in CDP-ethanolamine pathway activity at the *SELENOI*/EPT1-catalysed step. Western blots in yeast showed SELENOI/EPT1and the mutant version exhibited a projected molecular weight of 46 kDa and were detected at a similar level (Fig. 1M).

Subcellular lipidome imbalance has been shown to play an important role in the mechanisms underlying motor neuron diseases (Rickman *et al.*, 2020). Numerous genes involved in lipid metabolism have already been linked to HSP (Ahmed *et al.*, 2017). *PCYT2* and *SELENOI*/EPT1 are among the recently characterized HSP-associated genes and are currently reported only in a few families. *PCYT2* has been reported in six independent families presenting with a phenotypic spectrum ranging from pure to complex forms of HSP (Vaz *et al*., 2019; Vélez-Santamaría *et al.*, 2020) (Table 1). Impaired vision with nystagmus and seizures were among the frequent symptoms in reports of *PCYT2*. Variable features have included hearing loss, bilateral cataracts and ataxia. While brain MRI was unremarkable in one *PCYT2* case with pure HSP, other cases showed progressive, non-extensive T_2_-weighted white matter hyperintensities with cerebral or cerebellar atrophy.

**Table I:** Summary of genetic ﬁndings and clinical features of *SELENOI*/EPT1 and *PCYT2* cases

Family	This report	Ahmed *et al.*, 2017	Horibata *et al.*, 2018	This report	Vaz *et al*., 2019	Vélez-Santamaría *et al.*, 2020
**General information**						
Patient	Case 2	4 patients	1 patient	Case 1	5 patients	2 patients
*SELENOI/PCYT2* variants	*SELENOI* c.134C>T (p.Pro45Leu)	*SELENOI* c.335G>C (p.Arg112Pr)	*SELENOI* c.732-2A>G	*PCYT2* c.1112+1G>A; c.743T>A, p.(Val230Glu)	*PCYT2* c.920C>T, p.(His244Tyr)/ c.730C>T, p.(Pro307Leu); c.1129C>T, p.(Arg377Ter)	*PCYT2* c.957G>C, p.(Lys319Asn); c.907delG, p.(Val303Ter)/ c.1129C>T, p.(Arg377Ter)
Age (years)/sex	5/M	1.9–15/3M, 1F	4/M	26/F	2.5–20/4M, 1F	7 and 46/2M
Consanguinity	+	+	+	–	+ (4)	+ (1)
**Development**						
Global DD	+ (S)	+	+(S)	–	+ (5), 1S	+ (1)
ID	+ (P)	+	+	–	+ (5), 2P	+ (1S)
Poor growth	+ (S)	+	+	+	+	+ (1S)
Speech impairment	+ (S)	+ (3)	+	+^a^	+	–
Regression	–	+ (2)	–	–	+	–
**HSP-associated symptoms**						
Progressive microcephaly	+ (S)	+(2)	+	–	+(1)	–
Dysmorphic features	+FD	(4) CP, HP, BU	HP, BU	–	−(5)	+ (1)
Seizures	GTCS	–	TCS	+	TCS, FS	+ (1)
Visual impairment	+	+(2)	+	+	+(4)	+ (1)
Sensorineural deafness	–	–	+	+	+ (1)	–
Oculomotor abnormalities	+TU	–	ER	–	–	–
Peripheral neuropathy	NA	+ (1)	NA	–	NA	NA
Cerebellar ataxia	–	–	–	+	+ (1)	+ (1)
Bilateral cataract	+	–	–	+	–	+ (1)
Scoliosis	+	–	–	–	–	–
**Neurological examination**						
Nystagmus	–	–	+	+	+(4)	+ (1)
Dysphagia	+	–	–	–	–	–
Spasticity UL, LL	+ (LL, UL)	+(3 LL), 1 (UL, LL)	+ (UL, LL)	–	+ (LL)	+ (1 LL), (1 UL, LL)
Hyperreflexia	+	+	+	+	+	+
Hypotonia	–	–	Truncal	–	–	–
Joint contractures	+	NA	+	–	NA	–
**Investigations**						
Brain MRI	WMH (S), CA, CRA, BSA	WMH	WMH (S), CA, CRA, TCC	CRA	WMH, CA, CRA	WMH (1)
Ocular examination	NA	RP (2), CRD (1)	Absent VEP; normal ERG	SRD	OA (1)	OA (1)

BS = brainstem atrophy; BU = bifid uvula; CA = cerebral atrophy; CP = cleft palate; CRA = cerebellar atrophy; CRD = cone-rod dysfunction; DD = developmental delay; ER = eye roving; ERG = electroretinography; F = female; FD = facial dysmorphism; FS = focal seizures; GTCS = generalized tonic-clonic seizures; HP = high arched palate; HSP = hereditary spastic paraplegia; ID = intellectual disability; LL = lower limbs; M = male; NA = not available; OA = optic atrophy; P = profound; RP = retinitis pigmentosa; S = severe; SRD = severe retinal dystrophy; TCC = thin corpus callosum; TCS = tonic-clonic seizures; TU = tonic upgaze; UL = upper limbs; VEP = visual evoked potentials; WMH = T_2_ white matter hyperintensity.

a Impaired word fluidity.

Case 1 presented with a complex and progressive phenotype partly overlapping with the previously reported *PCYT2-*related HSP cases including visual impairment, bilateral cataract, sensorineural deafness and seizures (Vaz *et al.*, 2019; Vélez-Santamaría *et al.*, 2020). In this case, we show that confirmed EOSRD could be the early manifestation and a dominant feature of *PCYT2*-associated disorder. In contrast with the previous *PCYT2* reports, where cerebellar ataxia was a part of complex HSP in two affected individuals, Case 1 did not express spastic paraplegia, and severe cerebellar ataxia was the sole locomotor feature. This is not surprising, as various genes including those involved in phospholipid metabolism have been shown to present with HSP at one end of the disease continuum and ataxia at the other. Because of the overlapping phenotypes and shared disease mechanisms, these genes have been proposed to cause the ataxia-spasticity disease spectrum (ASS) (Synofzik and Schüle, 2017). It should be acknowledged, however, that the suggested association between the compound heterozygous *PCYT2* variants in Case 1 and ASS would have been strengthened if we had quantified the wild-type and misspliced mRNA resulting from the splice variant c.1112+1G>A or performed lipidomics studies.

*SELENOI*/EPT1 was first reported in *Brain* by Ahmed *et al.* (2017) in four siblings of Omani origin with a core phenotype similar to that of our case, albeit with a milder course (Table 1). These siblings were verbal and had gradually regressed after delayed achievements of developmental milestones. The spasticity was limited to the lower limbs in all but one sibling. They did not have clinically expressed seizures, but cleft palate, bifid uvula, retinal abnormalities and neurophysiological evidence of a demyelinating peripheral neuropathy were among their distinguishable signs. Ocular examination revealed pigmentary retinopathy and rod-cone dysfunction. The second family with *SELENOI*/EPT1-linked complex HSP was reported by Horibata *et al.* (2018) and had a spectrum of main features closer to those seen in our case, although with additional signs such as sensorineural deafness and roving eye movements (Table 1).

Although there were conspicuously overlapping core symptoms between Case 2 and the aforementioned *SELENOI*/EPT1 reports, the spectrum of the *SELENOI*/EPT1 phenotype seemed to be more severe in the present case. Features including severe growth failure, unachieved motor milestones and communication skills, severe dysphagia, congenital cataract and facial dysmorphism distinguished our case from the previously published *SELENOI*/EPT1 families. Cerebellar atrophy, brainstem atrophy and extensive hypomyelination were not observed in the family described by Ahmed *et al.* (2017), but these features were prominent in Case 2 and the report by Horibata *et al.* (2018). In addition, tonic upgaze was a peculiar feature in our report that could putatively be ascribed to the observed degree of hypomyelination, pontine/medullary and cerebellar atrophy (Hills *et al.*, 2013; Blumkin *et al.*, 2015).

Of note was the overlapping progressive phenotype between *PCYT2* and *SELENOI*/EPT1. Both expressed a comparable spectrum of associated symptoms and severity, suggesting the importance of the CDP-ethanolamine pathway in the mechanism of complex neurodegenerative disorders. *PCYT2* reports have unequivocally demonstrated how ascertaining more cases might expand the disease phenotype towards the milder end of the spectrum to pure HSP, and suggested the possibility of a predominant cerebellar ataxia phenotype with no spastic paraplegia as in Case 1.

Visual impairment was a common feature for almost all affected individuals with biallelic variants in *PCYT2* and *SELENOI*/EPT1. Ocular phenotyping revealed retinal pathology and optic atrophy, suggesting a particular susceptibility of retinal cells to the imbalance in CDP-ethanolamine and related pathways (Rickman *et al.*, 2020).

In summary, we have presented further families with biallelic variants in *PCYT2* and *SELENOI*/EPT1, thereby expanding the clinical spectrum of CDP-ethanolamine-related disorders. Additionally, we have highlighted the remarkable clinical overlap between *PCYT2* and *SELENOI*/EPT1 with retinal involvement being the common sign. Finally, we have drawn attention to the possibility of a broad ASS, which may result from defective PE biosynthesis. Awareness of a possible ASS phenotype and further reports will improve our understanding of the clinical spectrum and disease continuum of emerging CDP-ethanolamine-related disorders.

## Data availability

The data that support the findings of this study are available from the corresponding author, upon reasonable request.

## Web resources

The URLs for data presented herein are as follows:

http://www.ensembl.org/index.html

Genome Aggregation Database; http://gnomad.broadinstitute.org/

http://www.iranome.ir/

http://evs.gs.washington.edu/EVS/

http://www.ncbi.nlm.nih.gov/Omim

http://www.ncbi.nlm.nih.gov/pubmed/

https://www.ebi.ac.uk/interpro/protein/UniProt/Q9C0D9/

The 1000 Genomes Browser; http://browser.1000genomes.org/index.html

UCSC Human Genome Database; http://www.genome.ucsc.edu

Combined Annotation Dependent Depletion (CADD); http://cadd.gs.washington.edu/

NeurOmics: http://rd-neuromics.eu

https://www.rcsb.org/

## Supplementary Material

awaa442_Supplementary_Data
