# Corrigendum to: Defective phosphatidylethanolamine biosynthesis leads to a broad ataxia-spasticity spectrum

**DOI:** 10.1093/brain/awab022

**Published:** 2021-04-21

Rauan Kaiyrzhanov, Saskia Wortmann, Taryn Reid, Mohammadreza Dehghani, Mohammad Yahya Vahidi Mehrjardi, Bader Alhaddad, Matias Wagner, Marcus Deschauer, Isabell Cordts, J. Pedro Fernandez-Murray, Veronika Treffer, Zahra Metanat, Alan Pittman, Henry Houlden, Thomas Meitinger, Christopher Carroll, Christopher R. McMaster, Reza Maroofian. Defective phosphatidylethanolamine biosynthesis leads to a broad ataxia-spasticity spectrum. *Brain*. 2021;144(3);e30. doi:10.1093/brain/awaa442

The authors apologize for misspelling the last name of the author Alan Pittman. This has been corrected.

