# Supplementary material for: Defective phosphatidylethanolamine biosynthesis leads to a broad ataxia-spasticity spectrum
Source: Brain. 2021 Jan 17;144(3):e30. doi: 10.1093/brain/awaa442 (PMC8041042; doi:10.1093/brain/awaa442)
Supplement: awaa442_Supplementary_Data [file awaa442_supplementary_data.zip › brain-2020-01708-File004.pdf]

Supplementary Material for "Defective phosphatidylethanolamine biosynthesis leads to a broad ataxia-spasticity spectrum"

Supplementary Figures

Supplementary Figure 1: Sanger sequencing results for Case 1

Supplementary Figure 2: MRI of the spinal cord for Case 1

Supplementary Figure 3: cDNA analysis from fibroblast RNA in case 1

Supplementary Figure 4: The full unmodified image for blots used for Figure 1M

Supplementary Tables

Supplementary Table 1: Population frequencies and in silico pathogenicity predictions for *EPT1* and *PCYT2* variants found in this study

Supplementary methods

Supplementary methods 1: Materials and methods for human EPT1 in yeast expression studies

Supplementary methods 2: Protein modelling methods

Supplementary Video for Case 2

Supplementary references

Supplementary Figure 1: Sanger sequencing results for Case 1

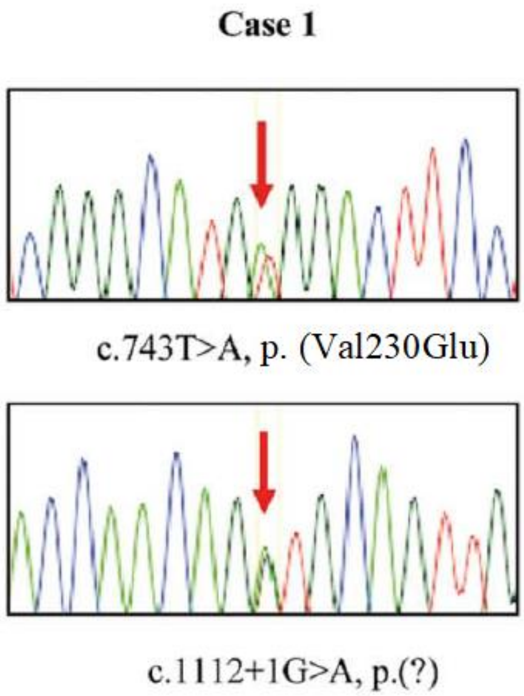

Supplementary Figure 2: MRI of the spinal cord for Case 1

T2 weighted image of axial thoracic segment at Th1. AP diameter is 4,9 mm. Normal diameter is 6.9 ± 1.6 mm (Frostell *et al.*, 2016).

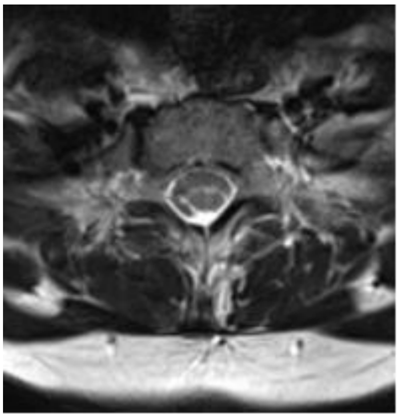

Supplementary Figure 3: cDNA analysis from fibroblast RNA in case 1

Supplementary image of the gel electrophoresis used for Figure 1 revealing two forms of PCYT2 splice products of 226 and 347 bp as a consequence of the variant c.1112+1G>A in Case 1. The experiment was repeated three times using different PCR conditions. Grey indicates that Orange indicates that Q-Solution (Quiagen) was added, yellow the addition of MgCl2 and orange that no additional PCR enhancers were used.

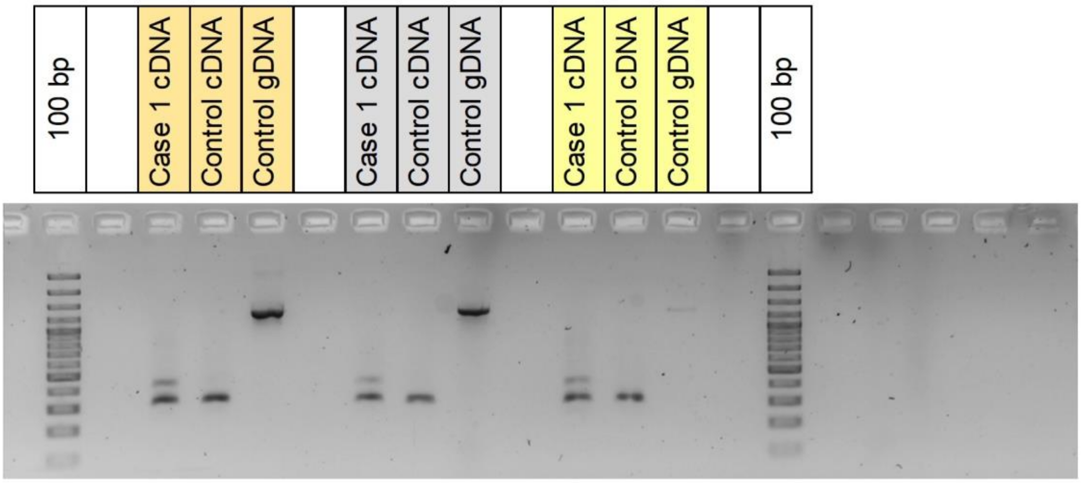

Supplementary Figure 4: The full unmodified image for blots used for Figure 1M

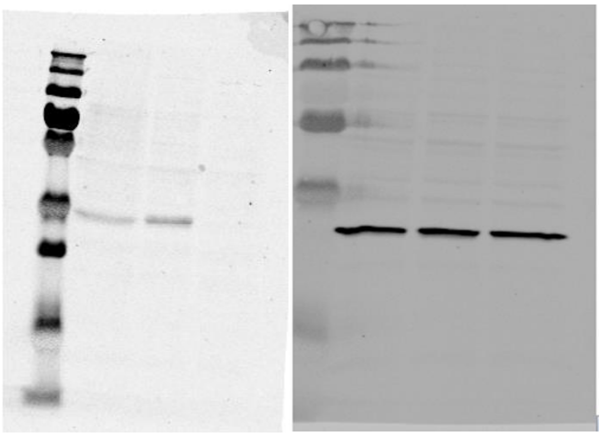

EPT1 **Dpm1**

The following commercial antibodies were used which undergone quality control testing by the vendor and have been used in numerous publications:
Yeast Dpm1 monoclonal antibody (5C5A7) - ThermoFisher - Catalog # A-6429. DDK epitope mouse monoclonal antibody - for detection of EPT1 with C-terminal DDK epitope tag - Origene - catalogue # TA180144.

Supplementary Table 1: Population frequencies and in silico pathogenicity predictions for *PCYT2* and *EPT1* variants found in this study

	**PCYT2**<br>**c.(1112+1 G>A)**	**PCYT2**<br>**c.(743 T>A),**<br>**p.(Val230Glu)**	**EPT1**<br><br>**c.134C>T, p.(Pro45Leu)**
**gnomAD**	absent	1 hete-<br>rozygous allele/<br>251.240 alleles	absent
**Iranom and GME**	absent	absent	absent
**Queen Square Genome database (16 000 exomes)**	absent	absent	absent
**The Munich database**	absent	absent	absent
**CADD PHRED score**	-	32	26.9
**GERP ++RS**	-	4.77	5.96
**SIFT score**	-	0<br>(deleterious)	0<br>(deleterious)
**PolyPhen score**	-	0.987<br>(probably damaging)	0.998<br>(probably damaging)
**MutTast score**	-	1<br>(disease causing)	1<br>(disease causing)
**PROVEAN**	-	-4.37<br>(damaging effect)	-9.89<br>(damaging effect)
**FATHMM PRED**	-	DDDD<br>(D - deleterios)	T<br>(T- tolerated)
**LRT PRED**	-	deleterios	deleterios
**MetaLR score**	-	0.9<br>(deleterious)	0.69<br>(deleterious)
**MutPred score**	-	0.87<br>(deleterious)	0.72<br>(deleterious)
**MutationAssessor score**	-	3.53<br>(deleterious)	3.8<br>(deleterious)
**REVEL score**	-	0.97<br>(likely disease causing)	0.74<br>(likely disease causing)

Supplementary methods 1: Materials and methods for human *EPT1* in yeast expression studies

Open reading frames expressing wild-type human SELENOI/EPT1, and the *EPT1*(pP45L) patient-derived mutant, were subcloned into the Saccharomyces cerevisiae expression vector p416-GPD allowing for constitutive expression from the GPD1 promoter. The TGA codon

encoding selenocysteine at amino acid 387 of EPT1 was changed to a cysteine encoding TGT codon by site-directed mutagenesis. The open reading frames contained a 3' extension encoding Myc and DDK tags to enable detection by western blot. Plasmids containing the wild-type and the mutant allele of human SELENOI/EPT1 were transformed into the yeast strain HJ091 (ept1-1 cpt1::LEU2). This yeast strain is devoid of endogenous ethanolaminephosphotransferase activity (Henneberry and McMaster, 1999; Henneberry *et al.*, 2000). Yeast cells containing the *SELENOI/ EPT1* expression plasmids were grown to mid-log phase and cells were incubated with radiolabeled ethanolamine for 1 hr. Cells were subjected to phase separation and lipids and aqueous metabolites were separated by thin layer chromatography as described previously (Henneberry and McMaster, 1999). Radiolabeled bands corresponding to CDP-ethanolamine pathway metabolites were scraped into vials and the level of radioactivity was determined by scintillation counting. Whole cell extracts were prepared from mid-log phase cells and fractionated into soluble and determine EPT1 protein expression, with Pgk1 (cytosolic fraction) and Dpm1 (membrane fraction) used as loading controls.

Supplementary methods 2: Protein modelling methods

Using the PCYT2 4XSV structure from the RCSB database, MOE version 2019.0102 and the Amber10:EHT force field were used to generate structures of the wild type and p.(Val230Glu) variant using energy minimized side chains. Molecular dynamics simulations were 500 ps equilibration and 1500 ps production with and without ligand. The substrate binding sites were docked using Site Finder based on p.(His226X227Glg228His229) locations.

Supplementary video for Case 2

Video of Case 2 showing tonic upgaze, feeding tube, upper limb spasticity and contractures, and feet deformities.

Supplementary references

Frostell A, Hakim R, Thelin EP, et al A review of the segmental diameter of the healthy human spinal cord. FrontNeurol., 2016 7:238–238.

Henneberry AL and McMaster CR. Cloning and expression of a human choline/ethanolaminephosphotransferase: synthesis of phosphatidylcholine and phosphatidylethanolamine. Biochem. J., 1999; 339: 291–8.

Henneberry AL, Wistow G, McMaster CR. Cloning, genomic organization, and characterization of a human cholinephosphotransferase. J. Biol. Chem., 2000; 275: 29808–15.
